# A Systematic Review and Meta-Analysis of Pharmacogenetic Studies in Patients with Chronic Kidney Disease

**DOI:** 10.3390/ijms22094480

**Published:** 2021-04-25

**Authors:** Maria Tziastoudi, Georgios Pissas, Georgios Raptis, Christos Cholevas, Theodoros Eleftheriadis, Evangelia Dounousi, Ioannis Stefanidis, Theoharis C. Theoharides

**Affiliations:** 1 Department of Nephrology, Faculty of Medicine, School of Health Sciences, University of Thessaly, 41110 Larissa, Greece; gpissas@msn.com (G.P.); teleftheriadis@med.uth.gr (T.E.); stefanid@med.uth.gr (I.S.); 2 Protypo Bioiatriko Laboratory, 41110 Larissa, Greece; raptislab@gmail.com; 3 AHEPA Hospital, First Department of Ophthalmology, Faculty of Health Sciences, School of Medicine, Aristotle University of Thessaloniki, 54621 Thessaloniki, Greece; ccholevas@hotmail.com; 4 Department of Nephrology, Faculty of Medicine, School of Health Sciences, University of Ioannina, 45110 Ioannina, Greece; edounous@uoi.gr; 5 Department of Immunology, Tufts University School of Medicine, Boston, MA 02155, USA; theoharis.Theoharides@tufts.edu

**Keywords:** genetic association, chronic kidney disease, meta-analysis, pharmacogenetics, systematic review

## Abstract

Chronic kidney disease (CKD) is an important global public health problem due to its high prevalence and morbidity. Although the treatment of nephrology patients has changed considerably, ineffectiveness and side effects of medications represent a major issue. In an effort to elucidate the contribution of genetic variants located in several genes in the response to treatment of patients with CKD, we performed a systematic review and meta-analysis of all available pharmacogenetics studies. The association between genotype distribution and response to medication was examined using the dominant, recessive, and additive inheritance models. Subgroup analysis based on ethnicity was also performed. In total, 29 studies were included in the meta-analysis, which examined the association of 11 genes (16 polymorphisms) with the response to treatment regarding CKD. Among the 29 studies, 18 studies included patients with renal transplantation, 8 involved patients with nephrotic syndrome, and 3 studies included patients with lupus nephritis. The present meta-analysis provides strong evidence for the contribution of variants harbored in the *ABCB1*, *IL-10*, *ITPA*, *MIF*, and *TNF* genes that creates some genetic predisposition that reduces effectiveness or is associated with adverse events of medications used in CKD.

## 1. Introduction

Chronic kidney disease (CKD) continues to constitute a global health burden. It is known that CKD elevates the risk of cardiovascular disease, kidney failure, and other complications [1,2,3]. According to the Kidney Disease Outcomes Quality Initiative (KDOQI) classification, CKD is defined as kidney damage or glomerular filtration rate (GFR) < 60 mL/min/1.73 m^2^ for 3 months or more, irrespective of the cause [4]. Although significant progress has been made in the treatment of nephrology patients with both conservative therapies and dialysis or transplantation, the emergence of drug-related problems such as ineffectiveness and side effects represents a major issue [5]. Pharmacogenetics could fill this gap [6].

Over the last 30 years, new drugs have been introduced to treat major kidney diseases, slow down the progression of CKD, and reduce the development of clinical complications associated with dialysis and kidney transplantation [7]. The use of different combinations of potent immunosuppressive drugs in transplant patients (calcineurin inhibitors, mammalian target of rapamycin inhibitors (mTORs), corticosteroids) have significantly improved the treatment of various renal disorders, and the short- and long-term pharmacological management of renal graft recipients [8].

In general, currently approved immunosuppressive drugs for maintenance therapy include calcineurin inhibitors (cyclosporine (CsA), tacrolimus (TAC)), mTOR inhibitors (sirolimus (SIR), everolimus), antiproliferatives (azathioprine (AZA) and mycophenolic acid (MPA)) and biologic drugs (belatacept) [9]. Differences between individuals regarding the efficacy and safety of immunosuppressive treatment are determined to some extent by genetic factors. For example, a common nonfunctional splicing variant, CYP3A5*3 (rs776746), determines TAC doses [10]. More specifically, patients with the CYP3A5*3/*3 genotype require less TAC to reach target concentrations compared with cytochrome P450 family 3 subfamily A member 5 (CYP3A5) CYP3A5*1 allele carriers [11]. Tacrolimus pharmacokinetic and pharmacodynamic variability is also attributed to ATP binding cassette subfamily B member 1 (*ABCB1*) variants: 1236C > T (rs1128503), 2677G > T/A (rs2032582), and 3435C > T (rs1045642) [12,13]. In addition, another example of the implication of pharmacogenetics in nephrology constitutes the thiopurine S-methyltransferase (*TPMT*) gene [14]. Many lines of evidence have reported that genetic variants located in the TPMT gene affect AZA metabolism and patients with low activity (10% prevalence) or absent activity (0.3% prevalence) are at risk of myelosuppression [15,16]. Among 20 variant alleles (*TPMT* *2-*18) identified to date, mutant alleles TPMT*2 and TPMT*3 explain more than 95% of defective gene activity [8,17].

“Adjusting” the dose of such drugs to the specific requirements of each patient to minimize toxicity while maintaining efficacy is a challenge in clinical nephrology. In an effort to provide the most comprehensive overview regarding the genetic contribution of pharmacogenes to the response to treatment of nephrology patients, we performed a systematic review and meta-analysis of available pharmacogenetic studies that included patients with CKD regardless of the primary cause of the disease.

## 2. Results

**Figure 1:**
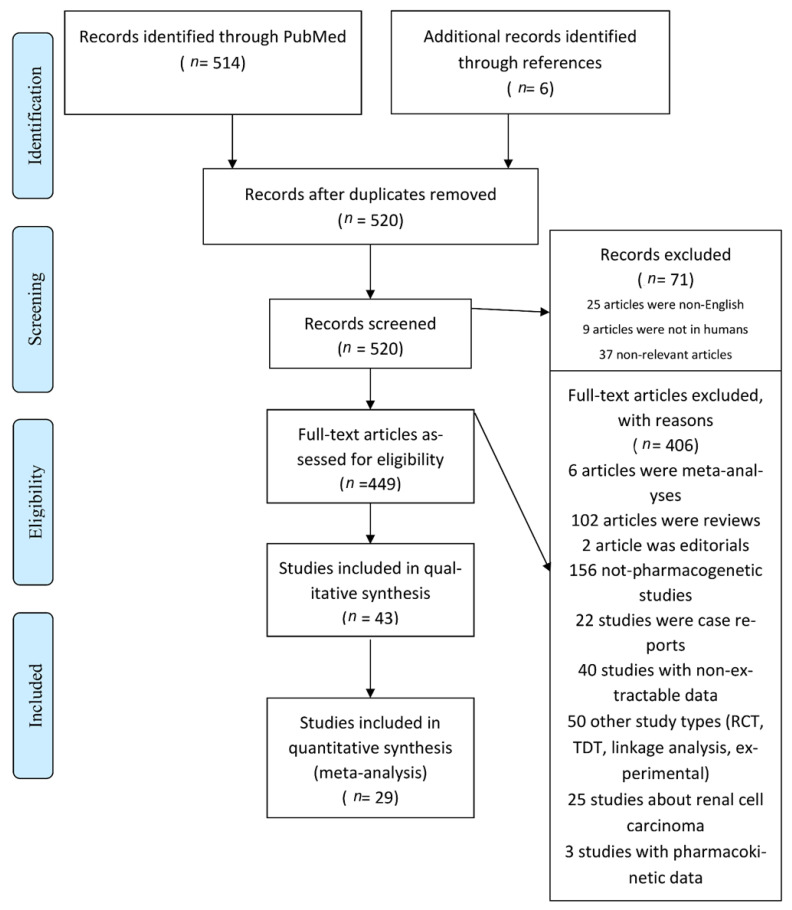
Flowchart of retrieved studies with reasons for exclusion.

A systematic review of the literature in the PubMed database identified 492 articles. After extensive study, 29 articles were included in the meta-analysis. Figure 1 shows the reasons for excluding articles. In total, 11 genes (*ABCB1*, *CYP2C9*, *CYP2C19*, *CYP3A5*, *IL-6*, *IL-10*, *ITPA*, *MIF*, *TGFB1*, *TNF*, *TPMT*) and 16 polymorphisms located in these genes were studied.

The characteristics of each study are listed in Table 1. The studies were conducted in various populations of different racial descent: 11 studies involved Caucasians, 14 studies recruited Asians, and 4 studies were conducted in ethnically mixed populations. Among the 29 studies, 18 studies included patients with renal transplantation, 8 recruited patients with nephrotic syndrome, and 3 studies included patients with lupus nephritis.

In total, 16 genetic polymorphisms were examined in two or more studies and, therefore, were meta-analyzed. Table 2, Table 3, Table 4, Table 5, Table 6 and Table 7 list the results of the meta-analyses that are indicative of the association of the respective polymorphism with the risk of side effects or non-response to medication in patients with CKD after calculating the odds ratio (OR) per genetic model.

More specifically, with regard to the *ABCB1* gene and the three polymorphisms harbored in it, the *ABCB1* 1236 C > T polymorphism was statistically significant in the studies with prednisolone (PRE) and mycophenolate (MMF). The *ABCB1* 2677 G > T polymorphism was also statistically significant in the analyses for PRE, whereas the *ABCB1* 3435 C > T polymorphism was statistically significant in the analyses for MMF and cyclosporine (CsA).

Regarding the genes encoding interleukins, the *IL-10* -592 C > A polymorphism in all genetic models and --819 C > T in the dominant and the additive model in the CsA analyses were statistically significant. Another statistically significant polymorphism was the ITPA 94 C > A polymorphism in the recessive model in azathioprine (AZA) analyses. In addition, a statistically significant polymorphism was the *MIF* -173 G > C polymorphism in PRE analyses in all genetic models. Statistically significant results were also obtained for the *TNF*-308 G > A polymorphism in the recessive and additive models in PRE analyses.

Regarding heterogeneity control, statistically significant heterogeneity was observed among the studies regarding the CYP2C19*2 polymorphism in the main analysis for cyclophosphamide (CYC): for the TPMT 1 vs. polymorphism, 3C, *MIF* -173 G > C, *Il-6* C-174G for PRE; for *TPMT* 1 vs. polymorphisms, 3C, *ABCB1* 1236 C > T, 2677 G > T, for CsA; for *TPMT* 1 vs. polymorphism 3C for AZA. For tacrolimus (TAC), a statistically significant heterogeneity was observed for polymorphisms *ABCB1* 2677 G > T and 3435C > T. Due to the statistically significant heterogeneity, the above results should be interpreted with caution, the majority of which are non-statistically significant.

On the existence of a difference in the estimated magnitude of genetic effects in large and small studies (or publication bias), which was assessed using the Egger test for funnel plot asymmetry and the Begg–Mazumdar test based on Kendall’s tau, the test was feasible in meta-analyses involving more than three studies. A statistically significant difference was observed between the *MIF* -173 G > C polymorphism studies in the PRE analysis.

## 3. Discussion

The present systematic review and meta-analysis provides the first comprehensive overview of pharmacogenetics studies in CKD regardless of the primary cause of the disease or the treatment. Although the term CKD is a very broad term, only 29 studies were included in the meta-analysis since many studies referred to pharmacokinetics without extractable genetic data. In total, 16 gene polymorphisms located in 11 different genes that were examined in 29 studies were included in the meta-analysis. The key finding of our meta-analysis was that variants *ABCB1* (1236 C > T, 2677 G > T, 3435 C > T), *IL-10* (-592 C > A, -819 C > T), *ITPA* (94 C > A), *MIF* (-173 G > C), and *TNF* (-308 G > A) gave significant results, suggesting the contribution of these loci to different responses to treatment in patients with CKD.

However, only *TPMT* has been included in the table of pharmacogenetics biomarkers in drug labeling of the U.S. Food and Drug administration (FDA) for the treatment of AZA [47]. More specifically, homozygous *TPMT*-deficient patients experience severe myelosuppression. For the other variants, the results are not so robust.

Most studies in the present systematic review are included in the meta-analysis of *ABCB1* variants [25,26,29,33,34,35,37,38,39,41,45]. These studies included a variety of treatments such as PRE, steroids, CsA, TAC, AZA, sirolimus (SIR), and MMF. It is noteworthy to be mentioned that no study with biologicals was included in the meta-analysis. Regarding calcineurin inhibitors, the effects of *ABCB1* 3435C > T, 1236C > T, and 2677G > T/A SNPs on the pharmacokinetics of CsA and TAC remain uncertain, with conflicting results. Genetic linkage between these three genotypes suggests that the pharmacokinetic effects are complex and unrelated to any *ABCB1* polymorphism. In contrast, it is possible that these polymorphisms may exert a small but combined effect. Any effect is likely to be in addition to the effects of *CYP3A5* 6986A > G SNP [12].

With regard to the *CYP3A5* 6986A > G variant, eight studies [23,25,34,36,40,41,44,45] included patients under treatment with pulse CYC, steroids, calcineurin inhibitors, and AZA/SIR. In contrast to CsA, a strong relationship between the CYP3A5 6986A > G SNP and TAC pharmacokinetics was demonstrated in kidney, heart, and liver transplant recipients, as well as in healthy volunteers [12]. Several recent studies have reported an approximate halving of the TAC C_0_/dose and doubling of the tacrolimus dose requirements in *CYP3A5* expressers compared to that in *CYP3A5* non-expressers [43,44,48,49,50,51,52].

However, studies with a small number of patients may be responsible for many conflicting results to date. The low frequency of some alleles, such as CYP3A4*1B allele, may not have been sufficient in many cases to detect a difference. In addition, the influence of ethnicity may play a role, as mutated genotypes are often more common in specific ethnic groups. However, even in the same ethnic group, for example in Caucasians, the frequencies of the studied polymorphisms differ. For instance, Caucasians present a minor allelic frequency around 50% regarding the *ABCB1* 1236C > T polymorphism, whereas the studied *TPMT* allele frequency polymorphisms range from 0.2–5.5% in Caucasians. Although the genotype itself, rather than the underlying ethnicity, should theoretically detect any differences, it is possible that indeterminate genetic differences (for example, co-inherited SNPs) among Africans, Caucasians, and Asians contribute to significant variables. In addition, the associations presented in these meta-analyses resulted from pooling a relatively small number of studies and patients with large heterogeneity between studies. Furthermore, the impact of effect modifiers such as age and the pre-treatment cytogenetic and molecular genetic findings was not considered as the individual studies did not provide the relevant data. Indeed, we have not included the analyses of interactions of age and comorbidity in the meta-analysis because these details were not included in the available data. It would be very interesting if future pharmacogenetic studies included this type of data in the analysis. The present systematic review and meta-analysis included studies that varied in terms of treatment and primary cause of CKD, as well as racial descent. Thus, the results should be interpreted with caution. Future studies with more homogenous studies will shed light on the pharmacogenetics in CKD. Thus, lack of significant association in the remaining gene variants does not exclude the possibility of an association.

Last but not least, epigenetic changes in drug metabolizing enzymes, nuclear receptors, and transporters are associated with individual drug responses and acquired multidrug resistance [53]. Consequently, pharmacoepigenetics could provide an explanation for why patients with the same genotype respond differently to therapy with a specific medication. Unrelated to epigenetics, inflammation can significantly influence the extent of CYP suppression, thus contributing to intra- and interindividual variability to drug exposure [54].

## 4. Materials and Methods

In order to clarify the contribution of the genetic background of CKD patients to the response to medications, a systematic review and meta-analysis of the pharmacogenetic studies reported in CKD patients was performed. The meta-analysis included studies published in English that are indexed in the PubMed database after a search with the terms (“pharmacogenetics” or “pharmacogenomics” or “response” or adverse effects” or “polymorphism” or “treatment”) AND (chronic kidney disease or nephrology or nephropathy or “kidney disease” or “glomerulonephritis”), accessed on 3 August 2020. In addition, all the references cited in the studies as well as the published meta-analyses that are relevant to the topic were also reviewed for any studies not indexed in PubMed. Unpublished data were not requested from any author.

The inclusion criteria that studies had to meet were: (a) included patients with CKD who did not respond to treatment or patients with CKD who had side effects due to medication (non-responders); (b) included patients with CKD who responded to treatment or patients with CKD who had no side effects due to medication (responders); (c) provided complete genotypic data by genotype for both responders to treatment and non-responders or allele frequencies, excluding studies that presented merged genotypic data.

Case reports, editorials, review articles, and publications with other study designs, such as family-based studies, were excluded. In studies with overlap, the most recent and largest study with data was included in the meta-analysis. Only studies using validated genotyping methods were considered. The eligibility of the studies was assessed independently by two researchers, the results were compared and any disagreement was resolved.

From each study, the following information was extracted: first author, year of publication, nationality of the study population, demographics, sample matching, and genotypic data of respondents and non-responders.

The association between genotype distribution and response to medication was examined using the dominant, recessive, and additive inheritance models. For all associations, the odds ratios (OR) with the corresponding 95% confidence intervals (CI) were recorded. A pooled OR was calculated based on the individual ORs. The threshold for meta-analysis was two studies per polymorphism. The pooled OR was calculated using fixed effects (FE) (Mantel–Haenszel) and random effects (RE) (DerSimonian and Laird) models. The random effects model assumes a genuine diversity in the results of the various studies and incorporates it into the variance calculations between studies. Heterogeneity between studies was tested using Cochran’s Q statistic (considered statistically significant at *p* < 0.10). Heterogeneity was quantified by measuring I^2^ (I^2^ = (Q − df)/Q), which is independent of the number of studies included in the meta-analysis. We also tested for small study effects with the Egger test and the Begg–Mazumdar test based on Kendall’s tau. Cumulative meta-analysis and retrospective meta-analysis were performed for each polymorphism to assess the trend of pooled OR over time.

For each study, we examined whether controls confronted with Hardy–Weinberg equilibrium (HWE) predicted genotypes using Fisher’s exact test. Finally, subgroup analyzes were performed based on ethnicity.

## 5. Conclusions

In conclusion, there is strong evidence that variants in the *ABCB1*, *IL-10*, *ITPA*, *MIF*, and *TNF* genes are related to poor response and/or adverse drug reactions in patients with CKD. Future studies would be required to confirm the results of the present meta-analysis, and an appropriate computer program could help guide the selection of the best drugs and doses.

## Figures and Tables

**Table 1:** Demographic characteristics of included studies.

Author (Year of Publication)	Ethnicity	Drug	Phenotype or Trait	Gene	Polymorphism (Rs Number)	*N*	Selection Criteria of Non-Responders	Responders	*N*	Selection Criteria of Responders
Xiong, 2010 [18]	East Asians	AZA	Kidney transplant recipients	*ITPA*	94C > A (rs1127354)	35	Hematotoxicity and/or hepatotoxicity and/or GI toxicity and/or flu-like symptoms	Renal transplants, AZA treatment present or previously	120	No adverse drug reactions
Kurzawski, 2009 [19]	Caucasians	AZA	Renal transplant recipients	*TPMT*	*1 vs. *2,*3A,*3C	108	Leucopenia and/or Hepatotoxicity	Renal transplants, AZA treatment previously	48	No adverse drug reactions
				*ITPA*	94C > A (rs1127354)					
Wang, 2008 [20]	Caucasians	TAC, MMF, PRE	Kidney transplant recipients (no antiviral, anticancer, or other leucopenia-causing medication)	*IMPDH1*	898G > A	60	Leucopenia	Renal transplants	129	No adverse drug reactions
				*IMPDH1*	rs2288550					
				*IMPDH1*	1552G > A					
Xin, 2009 [21]	East Asians	AZA, CsA, PRE	Renal transplant recipients	*TPMT*	*1 vs. *3C	30	Hematotoxicity and/or hepatotoxicity	Renal transplants	120	No adverse drug reactions
Vannaprasaht, 2009 [22]	Asians	AZA, PRE, CNIs	Kidney transplant recipients	*TPMT*	*1 vs. *3C	22	Myelosuppression	Renal transplants	117	No adverse drug reactions
Takada, 2004 [23]	Caucasians	pulse cyclophosphamide	Lupus nephritis	*CYP2C19*	CYP2C19*2 (rs4244285)	28	Development of premature ovarian failure	Patients with lupus nephritis	20	No adverse drug reactions
				*CYP2C9*	CYP2C9*2 (rs1799853)					
				*CYP3A5*	CYP3A5*3 (rs776746)					
Ngamjanyaporn, 2011 [24]	Asians	cyclophosphamide	SLE	*CYP2C19*	*1 vs. *2 (rs4244285)	36	Ovarian toxicity	Patients with systemic lupus erythematosus	35	No adverse drug reactions
Chiou, 2012 [25]	Asians	PRE	Idiopathic NS	*CYP3A5*	6986A > G (rs776746)	16	Steroid resistant NS	Patients with NS	58	Steroid sensitive NS
				*ABCB1*	C1236T (rs1128503)					
				*ABCB1*	G2677T (rs2032582)					
				*ABCB1*	G2677A (rs2032582)					
				*ABCB1*	C3435T (rs1045642)					
Youssef, 2013 [26]	Mixed	PRE	Idiopathic NS	*ABCB1*	C1236T (rs1128503)	46	Steroid non-responders	Patients with INS	92	Steroid responders
				*ABCB1*	G2677T/A (rs2032582)					
				*ABCB1*	C3435T (rs1045642)					
Sadeghi-Bojd, 2019 [27]	Asians	steroids	Idiopathic NS	*MIF*	-173G > C (rs755622)	27	Steroid resistant	Patients with NS	107	Steroid responders
Luo, 2013 [28]	East Asians	CsA	Gingival overgrowth in renal transplant recipients	*IL-10*	-1082A > G	122	With gingival overgrowth	Renal transplants	80	Without gingival overgrowth
				*IL-10*	-819C > T					
				*IL-10*	-592C > A					
Choi, 2011 [29]	East Asians	steroids	Idiopathic NS	*ABCB1*	1236C > T (rs1128503)	69	Steroid non-responders	Patients with NS	101	Steroid responders
				*ABCB1*	2677G > T (rs2032582)					
				*ABCB1*	2677G > A (rs2032582)					
				*ABCB1*	3435C > T (rs1045642)					
				*MIF*	G-173C (rs755622)					
Berdeli, 2005 [30]	Mixed	steroids	Idiopathic NS	*MIF*	G-173C (rs755622)	77	Steroid non-responders	Patients with NS	137	Steroid responders
Swierczewska, 2014 [31]	Caucasians	steroids	Idiopathic NS	*MIF*	G-173C (rs755622)	41	Steroid non-responders	Patients with NS	30	Steroid responders
Babel, 2004 [32]	Caucasians	CsA+ TAC/PRE and ATG/anti-IL-2R antibody	Long-term renal transplants	*IL10*	A-1082G (rs1800896)	51	Type 2/steroid-induced DM	Renal transplants	207	No adverse drug reactions
				*TNFa*	A-308G (rs1800629)					
				*IL-6*	C-174G					
				*TGFB1 10*	C > T					
Singh, 2011 [33]	Asians	CsA	Rejection episodes in renal transplant recipients	*ABCB1*	1236 C > T (rs1128503)	49	Rejection episodes	Renal transplants	176	No rejection episodes
		CsA		*ABCB1*	2677 G > T (rs2032582)	72			176	
		CsA		*ABCB1*	3435 C > T (rs1045642)	70			176	
		TAC		*ABCB1*	1236 C > T (rs1128503)	46			29	
		TAC		*ABCB1*	2677 G > T (rs2032582)	46			29	
		TAC		*ABCB1*	3435 C > T (rs1045642)					
Santoro, 2011 [34]	Mixed	CsA and AZA/SRL or TAC and AZA/SRL	Renal transplant patients	*CYP3A5*	CYP3A5*3 (rs776746)	15	Biopsy-proven rejection episodes	Renal transplants	138	No biopsy-proven rejection episodes
				*ABCB1*	1236 C > T (rs1128503)	139			15	
				*ABCB1*	2677 G > T (rs2032582)	129			15	
				*ABCB1*	3435 C > T (rs1045642)	140			15	
Glowacki, 2011 [35]	Caucasians	TAC	Acute tubular necrosis/TAC tubular or vascular toxicity after renal transplantation	*ABCB1*	3435 C > T (rs1045642)	16	Acute tubular necrosis/TAC tubular or vascular toxicity	Renal transplants	187	No acute tubular necrosis/TAC tubular or vascular toxicity
Kuypers, 2010 [36]	Caucasians	calcineurin inhibitor	Calcineurin inhibitor-associated nephrotoxicity in renal allograft recipients	*CYP3A5*	CYP3A5*3 (rs776746)	51	Calcineurin inhibitor-associated nephrotoxicity	Renal allograft recipients	253	
Miura, 2008 [37]	East Asians	PRE and TAC and MMF	Acute rejection in renal transplant recipients	*ABCB1*	1236 C > T (rs1128503)	43	Acute rejection	Renal transplants	52	No acute rejection
				*ABCB1*	2677 G > T (rs2032582)					
				*ABCB1*	2677 G > A (rs2032582)					
				*ABCB1*	3435 C > T (rs1045642)					
Grinyo, 2008 [38]	Caucasians	CsA and MMF	Acute rejection after kidney transplantation	*ABCB1*	3435 C > T (rs1045642)	77	Biopsy-proven acute rejection	Renal transplants	160	No biopsy-proven acute rejection
				*ABCB1*	1236 C > T (rs1128503)					
				*ABCB1*	2677 G > T (rs2032582)					
				*ABCB1*	2677 G > A (rs2032582)					
				*IMPDH1*	G1320A					
				*IL-10*	C-592A (rs1800872)					
				*IL-10*	A-1082G (rs1800896)					
				*IL-10*	C-819T (rs3021097)					
				*TGF-b1*	C869T (rs1800470)					
Von Ahsen, 2001 [39]	Caucasians	CsA	Rejection episodes in stable renal transplant recipients	*ABCB1*	3435 C > T (rs1045642)	47	Rejection	Renal transplants	77	No rejection
Quteineh, 2008 [40]	Caucasians	TAC	Delayed allograft function in renal graft recipients	*CYP3A5*	CYP3A5*3 (rs776746)	77	Delayed graft function	Renal transplants	59	No delayed graft function
Qiu, 2008 [41]	East Asians	CsA	Rejection episodes in renal transplant recipients	*ABCB1*	1236 C > T (rs1128503)	6	Rejection	Renal transplants	97	No rejection
				*ABCB1*	2677 G > T/A (rs2032582)	6			97	
				*ABCB1*	3435 C > T (rs1045642)	6			97	
				*CYP3A5*	CYP3A5*3 (rs776746)	6			97	
Kagaya, 2010 [42]	Asians	MMF	Subclinical acute rejection after renal transplantation	*IMPDH*	rs2278293	21	Subclinical acute rejection		61	No subclinical acute rejection
				*IMPDH*	rs2278294					
Kurzawski, 2005 [43]	Caucasians	AZA	AZA-induced myelotoxicity in renal transplant recipients	*TPMT*	*1 vs. *2,*3A,*3C	67	AZA-induced myelotoxicity	Renal transplants	113	No adverse drug reactions
Kumaraswami, 2017 [44]	Asians	cyclophosphamide	Lupus nephritis	*CYP2C19*	CYP2C19*2 (rs4244285)	24	No response	Lupus nephritis patients	123	Complete and partial response
				*CYP2C9*	CYP2C9*2 (rs1799853)					
				*CYP3A5*	CYP3A5*3 (rs776746)					
Moussa, 2017 [45]	Mixed	steroids	Pediatric idiopathic nephrotic syndrome	*ABCB1*	C1236T (rs1128503)	10	Steroid non-responders	Idiopathic nephrotic syndrome	53	Steroid responders
				*ABCB1*	G2677A					
				*ABCB1*	C3435T (rs1045642)					
				*CYP3A5*	CYP3A5*3 (rs776746)					
Tripathi, 2008 [46]	Asians	glucocorticoids	Idiopathic nephrotic syndrome	*TNF-α*	A-308G (rs1800629)	35	Steroid resistant	Idiopathic nephrotic syndrome	115	Steroid sensitive
				*IL-6*	G174C (rs1800795)					

**Table 2:** Meta-analysis results regarding pulse cyclophosphamide.

*Drug*	Gene	Polymorphism	Rs Number	N of Studies	OR with 95% CI Fixed Effects	OR with 95% CI Random Effects	I^2^ (%)	*p*-Value for Q	Egger Test *p*-Value	Begg–Mazumdar *p*-Value
***Pulse cyclophosphamide***	*CYP2C9*	CYP2C9*2	rs1799853	2						
***All***										
***Dominant***					1.24 (0.20–7.90)	1.24 (0.20–7.90)	0	0.41	-	-
***Recessive***					1.89 (0.11–32.69)	1.89 (0.11–32.69)	0	0.52		
***Additive***					1.93 (0.11–33.45)	1.93 (0.11–33.45)	0	0.54		
Pulse cyclophosphamide	*CYP2C19*	CYP2C19*2 (G681A)	rs4244285	3						
*All*										
Dominant					1.07 (0.60–1.90)	0.81 (0.17–3.90)	86	0.001	-	-
Recessive					1.25 (0.34–4.63)	1.25 (0.34–4.63)	0	0.89		
Additive					1.36 (0.34–5.36)	1.36 (0.34–5.36)	0	0.48		
Caucasians				1			-	-		
Asians				2						
Dominant					1.88 (0.98–3.60)	1.88 (0.98–3.60)	0	0.50	-	-
Recessive					1.46 (0.33–3.67)	1.46 (0.33–3.67)	0	0.84		
Additive					2.06 (0.44–9.58)	2.06 (0.44–9.58)	0	0.94		
										
Pulse cyclophosphamide	*CYP3A5*	CYP3A5*3	rs776746							
All				2						
Dominant					0.67 (0.30–1.48)	0.67 (0.30–1.48)	0%	0.54	-	-
Recessive					0.90 (0.30–2.68)	0.90 (0.30–2.68)	0%	0.58	-	-
Additive					0.73 (0.17–3.08)	0.73 (0.17–3.08)	0%	0.32	-	-

**Table 3:** Meta-analysis results regarding prednisolone.

*Drug*	Gene	Polymorphism	Rs Number	N of Studies	OR with 95% CI Fixed Effects	OR with 95% CI Random Effects	I^2^ (%)	*p*-Value for Q	Egger Test *p*-Value	Begg–Mazumdar *p*-Value
*Prednizolone*										
All	*TPMT*	*1 vs. *3C		2						
Dominant					0.49 (0.18–1.37)	0.64 (0.01–50.02)	94.4%	<0.0001	-	-
Recessive					4 (0.08–202.85)	4 (0.08–202.85)	0%	>0.9999	-	-
Additive					4.5 (0.09–228.51)	4.5 (0.09–228.51)	0%	>0.9999	-	-
										
All	*CYP3A5*	CYP3A5*3	rs776746	2						
Dominant					2.38 (0.41–13.67)	2.38 (0.41–13.67)	0%	0.84	-	-
Recessive					**2.54 (1.03–6.22)**	**2.54 (1.03–6.22)**	0%	0.73	-	-
Additive					3.24 (0.54–19.51)	3.24 (0.54–19.51)	0%	0.80	-	-
All	*ABCB1*	C3435T	rs1045642	9						
Dominant					0.86 (0.63–1.18)	0.86 (0.63–1.18)	0%	0.61	0.62	0.48
Recessive					1.21 (0.86–1.70)	1.21 (0.86–1.70)	0%	0.76	0.72	0.76
Additive					0.97 (0.64–1.48)	0.97 (0.64–1.48)	0%	0.95	0.31	0.61
Caucasians	*ABCB1*	C3435T	rs1045642	2						
Dominant					1.02 (0.28–3.68)	1.05 (0.26–4.28)	14.7%	0.28	-	-
Recessive					2.02 (0.82–4.96)	2.05 (0.73–5.75)	23.6%	0.25	-	-
Additive					1.84 (0.46–7.32)	1.84 (0.46–7.32)	0%	0.68	-	-
Asians	*ABCB1*	C3435T	rs1045642	5						
Dominant					0.89 (0.62–1.28)	0.89 (0.62–1.28)	0%	0.83	0.24	0.48
Recessive					1.07 (0.66–1.75)	1.07 (0.66–1.75)	0%	0.86	0.82	0.48
Additive					1.01 (0.59–1.74)	1.01 (0.59–1.74)	0%	0.99	0.79	0.82
Mixed	*ABCB1*	C3435T	rs1045642	2						
Dominant					0.75 (0.39–1.44)	0.66 (0.19–2.31)	70.6%	0.07	-	-
Recessive					1.17 (0.68–2.02)	1.17 (0.68–2.02)	0%	0.36	-	-
Additive					0.76 (0.37–1.59)	0.76 (0.36–1.61)	3.8%	0.31	-	-
All	*ABCB1*	**C1236T**	**rs1128503**	9						
Dominant					1.29 (0.91–1.84)	1.31 (0.90–1.89)	5%	0.39	0.62	0.36
Recessive					**1.70 (1.22–2.38)**	**1.62 (1.10–2.40)**	20.4%	0.26	0.09	0.26
Additive					**1.63 (1.01–2.64)**	1.62 (0.95–2.76)	14%	0.32	0.72	0.76
Caucasians	*ABCB1*	C1236T	rs1128503	2						
Dominant					0.56 (0.21–1.52)	0.56 (0.21–1.52)	0%	0.38	-	-
Recessive					0.94 (0.33–2.63)	0.94 (0.33–2.63)	0%	0.65	-	-
Additive					0.63 (0.18–2.22)	0.63 (0.18–2.22)	0%	0.42	-	-
Asians	*ABCB1*	C1236T	rs1128503	5						
Dominant					1.42 (0.91–2.21)	1.48 (0.90–2.43)	7.6%	0.36	0.27	0.82
Recessive					**1.69 (1.11–2.60)**	1.58 (0.88–2.83)	37.1%	0.17	0.46	0.48
Additive					**1.90 (1.02–3.53)**	1.92 (0.88–4.19)	27.2%	0.24	0.94	0.82
Mixed	*ABCB1*	C1236T	rs1128503	2						
Dominant					1.55 (0.79–3.05)	1.55 (0.79–3.05)	0%	0.68	-	-
Recessive					2.17 (1.14–4.12)	2.06 (0.88–4.81)	39.3%	0.20	-	-
Additive					1.97 (0.76–5.12)	1.97 (0.76–5.12)	0%	0.46	-	-
Prednizolone	*ABCB1*	*G2677T*	rs2032582	*5*						
All										
Dominant					1.08 (0.60–1.93)	1.08 (0.60–1.93)	0%	0.83	0.43	0.23
Recessive					1.16 (0.67–2.01)	1.11 (0.48–2.57)	53.8%	0.07	0.72	0.08
Additive					1.34 (0.66–2.71)	1.34 (0.66–2.71)	0%	0.73	0.76	0.48
Caucasians	*ABCB1*	*G2677T*	rs2032582	*2*						
*Dominant*					1.42 (0.36–5.62)	1.42 (0.36–5.62)	0%	0.57	-	-
*Recessive*					0.64 (0.24–1.70)	0.62 (0.15–2.61)	53.5%	0.14	-	-
*Additive*					0.89 (0.19–4.14)	0.91 (0.16–5.23)	22.3%	0.26	-	-
*Asians*	*ABCB1*	*G2677T*	*rs2032582*	*3*						
*Dominant*					1.01 (0.53–1.93)	1.01 (0.53–1.93)	0%	0.63	-	-
*Recessive*					1.53 (0.78–3.00)	1.57 (0.55–4.47)	54.6%	0.11	-	-
*Additive*					1.49 (0.67–3.30)	1.49 (0.67–3.30)	0%	0.82	-	-
Prednizolone	*ABCB1*	G2677A	rs2032582							
All				5						
Dominant					1.21 (0.62–2.37)	1.30 (0.59–2.84)	21.1%	0.28	0.16	0.08
Recessive					1.64 (0.60–4.47)	1.64 (0.60–4.47)	0%	0.68	0.48	0.82
Additive					1.22 (0.38–3.91)	1.22 (0.38–3.91)	0%	0.55	0.23	0.23
Caucasians	*ABCB1*	G2677A	rs2032582	1						
*Asians*				4						
*Dominant*					1.07 (0.54–2.14)	1.08 (0.53–2.18)	2.9%	0.38	0.50	0.75
*Recessive*					1.39 (0.48–4.01)	1.39 (0.48–4.01)	0%	0.70	0.90	0.75
*Additive*					0.91 (0.26–3.13)	0.91 (0.26–3.13)	0%	0.76	0.49	0.33
										
Prednizolone	*MIF*	−173 G > C	rs755622							
All				*4*						
*Dominant*					1.56 (1.09–2.24)	1.28 (0.55–3.00)	80.6%	0.001	0.16	<0.0001
*Recessive*					2.90 (1.02–8.30)	2.88 (0.68–12.16)	45.3%	0.14	0.91	0.75
*Additive*					2.98 (1.03–8.63)	2.93 (0.54–15.99)	59.4%	0.06	0.92	0.75
										
Prednizolone	*IL-6*	*C-174G*	rs1800795							
*All*				2						
*Dominant*					0.82 (0.49–1.37)	0.82 (0.49–1.37)	0%	0.69	-	-
*Recessive*					0.80 (0.43–1.48)	0.32 (0.02–4.28)	82.8%	0.02	-	-
*Additive*					0.66 (0.31–1.40)	0.31 (0.02–3.76)	80.9%	0.02	-	-
										
Prednizolone	*TNF*	G-308A								
*All*				2						
*Dominant*					0.82 (0.49–1.38)	0.82 (0.49–1.38)	0%	0.35	-	-
*Recessive*					**0.12 (0.02–0.65)**	**0.12 (0.02–0.65)**	0%	0.38		
*Additive*					**0.12 (0.02–0.64)**	**0.12 (0.02–0.64)**	0%	0.38		

**Table 4:** Meta-analysis results regarding MMF.

*Drug*	Gene	Polymorphism	Rs Number	N of Studies	OR with 95% CI Fixed Effects	OR with 95% CI Random Effects	I^2^ (%)	*p*-Value for Q	Egger Test *p*-Value	Begg–Mazumdar *p*-Value
*MMF*	*ABCB1*	3435C > T	rs1045642							
*All*				2						
*Dominant*					**2.07 (1.09–3.94)**	**2.07 (1.09–3.94)**	**0%**	0.41	-	-
*Recessive*					1.43 (0.81–2.54)	1.27 (0.52–3.09)	46.3%	0.17	-	-
*Additive*					**2.25 (1.05–4.84)**	1.99 (0.64–6.22)	47.2%	0.17	-	-
*MMF*	*ABCB1*	1236C > T	rs1128503							
*All*				2						
*Dominant*					1.67 (0.93–3.00)	1.67 (0.93–3.00)	0%	0.51	-	-
*Recessive*					**1.89 (1.05–3.40)**	1.63 (0.52–5.11)	70.2%	0.07	-	-
*Additive*					**2.43 (1.17–5.04)**	2.13 (0.73–6.18)	33.9%	0.22	-	-
*MMF*	*ABCB1*	2677G > T	rs2032582							
*All*				2						
*Dominant*					**2.20 (1.16–4.17)**	**2.20 (1.16–4.17)**	0%	0.81	-	-
*Recessive*					1.79 (0.94–3.40)	1.37 (0.36–5.18)	66.2%	0.09	-	-
*Additive*					**2.92 (1.32–6.46)**	**2.77 (1.09–7.05)**	14%	0.28	-	-
*MMF*	*ABCB1*	2677G > A	rs2032582							
*All*				2						
*Dominant*					3.72 (0.72–19.22)	3.72 (0.72–19.22)	0%	0.50	-	-
*Recessive*					3.04 (0.22–42.65)	3.04 (0.22–42.65)	0%	0.75	-	-
*Additive*					4.14 (0.28–61.96)	4.14 (0.28–61.96)	0%	0.94	-	-

**Table 5:** Meta-analysis results regarding cyclosporine.

*Drug*	Gene	Polymorphism	Rs Number	N of Studies	OR with 95% CI Fixed Effects	OR with 95% CI Random Effects	I^2^ (%)	*p*-Value for Q	Egger Test *p*-Value	Begg–Mazumdar *p*-Value
Cyclosporine (CsA)	*TPMT*	1 vs. 3C								
All				2						
Dominant					0.49 (0.18–1.37)	0.64 (0.01–50.02)	94.4%	<0.0001	-	-
Recessive					4 (0.08–202.85)	4 (0.08–202.85)	0%	>0.9999	-	-
Additive					4.5 (0.09–228.51)	4.5 (0.09–228.51)	0%	>0.9999	-	-
CsA	*IL10*	−1082A > G								
All				3						
Dominant					0.75 (0.49–1.14)	0.76 (0.42–1.37)	48.1%	0.15	-	-
Recessive					1.11 (0.70–1.77)	1.11 (0.70–1.77)	0%	0.93	-	-
Additive					1.04 (0.59–1.85)	1.04 (0.59–1.85)	0%	0.59	-	-
CsA	*IL10*	−819C > T								
All				2						
Dominant					**1.72 (1.09–2.72)**	**1.72 (1.09–2.72)**	0%	0.33	-	-
Recessive					**1.90 (1.12–3.24)**	2.30 (0.82–6.40)	61.9%	0.11	-	-
Additive					**2.70 (1.43–5.10)**	**2.70 (1.43–5.10)**	0%	0.56	-	-
CsA	*IL10*	−592C > A								
All				2						
Dominant					**1.67 (1.07–2.60)**	**1.67 (1.04–2.70)**	13.5%	0.28	-	-
Recessive					**1.93 (1.16–3.22)**	2.17 (0.91–5.19)	57.6%	0.12	-	-
Additive					**2.79 (1.52–5.13)**	**2.79 (1.52–5.13)**	0%	0.49	-	-
CsA	*TGFB1*	C869T (P10L)								
All				2						
Dominant					0.80 (0.47–1.37)	0.80 (0.47–1.37)	0%	0.67	-	-
Recessive					0.68 (0.44–1.05)	0.68 (0.44–1.05)	0%	0.49	-	-
Additive					0.66 (0.36–1.19)	0.66 (0.36–1.19)	0%	0.94	-	-
CsA	*ABCB1*	1236C > T	rs1128503							
All				4						
Dominant					0.91 (0.59–1.40)	0.82 (0.32–2.14)	71%	0.02	0.88	0.75
*Recessive*					1.14 (0.72–1.80)	1.00 (0.38–2.60)	70.5%	0.02	0.68	0.75
*Additive*					1.04 (0.60–1.80)	0.91 (0.23–3.58)	77.1%	0.00	0.84	0.75
*CsA*										
*All*				3						
*Dominant*					0.88 (0.55–1.38)	0.85 (0.24–3.01)	85.7%	0.001	-	-
*Recessive*					1.03 (0.63–1.69)	1.33 (0.31–5.80)	83.7%	0.00	-	-
*Additive*					0.97 (0.54–1.75)	1.32 (0.17–10.44)	88.9%	0.0001	-	-
*CsA*	*ABCB1*	3435 C > T	rs1045642							
*All*				5						
*Dominant*					1.02 (0.67–1.54)	1.02 (0.55–1.90)	50.6%	0.09	0.94	0.48
*Recessive*					1.47 (1.01–2.16)	1.47 (1.01–2.16)	0%	0.84	0.64	0.82
*Additive*					1.33 (0.81–2.18)	1.37 (0.71–2.67)	33.7%	0.20	0.70	0.48
*CsA*										
*All*				3						
*Dominant*					0.44 (0.09–2.16)	0.44 (0.09–2.16)	0%	0.999	-	-
*Recessive*					0.98 (0.53–1.82)	0.98 (0.53–1.82)	0%	0.78	-	-
*Additive*					0.48 (0.09–2.40)	0.48 (0.09–2.40)	0%	0.97	-	-

**Table 6:** Meta-analysis results regarding azathioprine.

*Drug*	Gene	Polymorphism	Rs Number	N of Studies	OR with 95% CI Fixed Effects	OR with 95% CI Random Effects	I^2^ (%)	*p*-Value for Q	Egger Test *p*-Value	Begg–Mazumdar *p*-Value
*Azathioprine*	*TPMT*	1 vs. 3C								
*All*				**4**						
*Dominant*					1.64 (0.83–3.26)	2.14 (0.22–21.08)	90.1%	<0.0001	0.75	0.33
*Recessive*					2.33 (0.24–22.55)	2.33 (0.24–22.55)	0%	0.99	0.80	>0.9999
*Additive*					2.78 (0.29–26.75)	2.78 (0.29–26.75)	0%	0.99	0.59	>0.9999
*Azathioprine*	*ITPA*	94C > A	rs1127354							
*All*				2						
*Dominant*					1.60 (0.84–3.06)	1.59 (0.81–3.14)	8.6%	0.30	-	-
*Recessive*					**21.82 (1.07–445.72)**	**21.82 (1.07–445.72)**	0%	>0.9999	-	-
*Additive*					10.19 (0.92–113.39)	10.19 (0.92–113.39)	0%	0.35	-	-

**Table 7:** Meta-analysis results regarding tacrolimus.

*Drug*	Gene	Polymorphism	Rs Number	N of Studies	OR with 95% CI Fixed Effects	OR with 95% CI Random Effects	I^2^ (%)	*p*-Value for Q	Egger Test *p*-Value	Begg–Mazumdar *p*-Value
*Tacrolimus*	*CYP3A5*	CYP3A5*3	rs776746							
*All*				**3**						
*Dominant*					**0.24 (0.08–0.69)**	**0.24 (0.08–0.69)**	0%	0.86	-	-
*Recessive*					0.88 (0.53–1.46)	0.88 (0.53–1.46)	0%	0.87	-	-
*Additive*					**0.25 (0.08–0.77)**	**0.25 (0.08–0.77)**	0%	0.91	-	-
*Tacrolimus*	*ABCB1*	1236C > T	rs1128503							
*All*				2						
*Dominant*					1.53 (0.62–3.81)	1.53 (0.62–3.81)	0%	0.54	-	-
*Recessive*					1.08 (0.52–2.21)	1.08 (0.52–2.21)	0%	0.54	-	-
*Additive*					1.48 (0.54–4.10)	1.48 (0.54–4.10)	0%	0.49	-	-
*Tacrolimus*	*ABCB1*	2677 G > T	rs2032582							
*All*				2						
*Dominant*					0.44 (0.17–1.10)	0.58 (0.07–4.61)	77.3%	**0.04**	-	-
*Recessive*					0.46 (0.21–1.03)	0.46 (0.21–1.03)	0%	0.66	-	-
*Additive*					**0.33 (0.12–0.91)**	0.40 (0.08–2.14)	56%	0.13	-	-
*Tacrolimus*	*ABCB1*	3435C > T	rs1045642							
*All*				3						
*Dominant*					0.76 (0.43–1.34)	0.66 (0.21–2.13)	73.7%	**0.02**	-	-
*Recessive*					1.47 (0.83–2.59)	1.24 (0.43–3.57)	69.4%	**0.04**	-	-
*Additive*					1.06 (0.53–2.12)	0.83 (0.20–3.47)	74.2%	**0.02**	-	-

## Abbreviations

Chronic kidney disease	CKD
Kidney Disease Outcomes Quality Initiative	KDOQI
Mammalian target of rapamycin inhibitors	mTORs
Cyclosporine	CsA
Tacrolimus	TAC
Sirolimus	SIR
Azathioprine	AZA
Mycophenolic acid	MPA
Mycophenolate	MMF
ATP binding cassette subfamily B member 1	*ABCB1*
cytochrome P450 family 2 subfamily C member 9	*CYP2C9*
cytochrome P450 family 2 subfamily C member 19	*CYP2C19*
cytochrome P450 family 3 subfamily A member 5	*CYP3A5*
interleukin 6	*IL-6*
interleukin 10	*IL-10*
inosine triphosphatase	*ITPA*
macrophage migration inhibitory factor	*MIF*
transforming growth factor beta 1	*TGFB1*
tumor necrosis factor	*TNF*
thiopurine S-methyltransferase	*TPMT*
